# Ce Doping Effects on the Hydrogen Sensing Properties of Graphene/SnO_2_-Based Sensors

**DOI:** 10.3390/ma17174382

**Published:** 2024-09-05

**Authors:** Zijie Jiao, Lingyun Wang, Xiaotong Xu, Jie Xiang, Shuiming Huang, Tao Lu, Xueling Hou

**Affiliations:** 1 School of Materials Science and Engineering, Shanghai University, Shanghai 200072, China; jiao_zj@163.com (Z.J.); wly18395354697@163.com (L.W.); xuxiaotong0908@163.com (X.X.); xj@kingmt.com.cn (J.X.); hshuiming@kingmt.com.cn (S.H.); 2 Shanghai King Material Technology Ltd., East Huiwang Road, Jiading, Shanghai 201815, China; jw@kingmt.com.cn

**Keywords:** hydrogen gas sensors, Ce-SnO_2_/SLG, doping, Ce, n–n heterojunction, p–n heterojunction

## Abstract

The development of a sensor capable of selectively detecting hydrogen levels in the environment holds immense importance for ensuring the safer utilization of hydrogen energy. In this study, a hydrogen sensor made of Ce-doped single-layer graphene (SLG)/SnO_2_ composite material was fabricated using a hydrothermal method. The study examined the impact of varying Ce doping concentrations on the hydrogen sensing capabilities of the SLG/SnO_2_ matrix. The results show that the SLG/SnO_2_ hydrogen sensor doped with 2 mol% Ce demonstrated optimal performance at a humidity of 20%. It operated most efficiently at 250 °C, with a response of 2.49, representing a 25.75% improvement over the undoped sample. The response/recovery times were 0.46/3.92 s, which are 54.9% shorter than those of the undoped sample. The enhancement in hydrogen sensitivity stems from the synergistic effect of Ce and SLG, which facilitates the coexistence of n–n and p–n heterojunctions, thereby increasing carrier mobility and refining grain structure. Analysis via X-ray photoelectron spectroscopy (XPS) reveals that Ce increases the material’s oxygen vacancy concentration, enhancing its hydrogen sensitivity. Ce-doped SLG/SnO_2_, with its robust hydrogen sensitivity, represents one of the leading candidates for future hydrogen gas sensors.

## 1. Introduction

In recent years, people have been advocating and committed to the development and utilization of green and renewable energy, and then applying it to various aspects of people’s lives [1]. Compared to other clean energy sources such as solar and wind energy that are limited by the application environment, hydrogen energy has excellent performance such as high combustion efficiency, long-term storage capacity, and low pollution, which enables the more efficient storage and transportation of hydrogen energy, more convenient use, and stable energy supply [2,3]. At present, hydrogen energy is widely used in various fields such as civil, industrial, transportation, power, and energy storage, as well as high-end fields such as aviation [4]. However, pure H_2_ is colorless, odorless, tasteless, and suffocating, making it difficult for humans to detect leaks when they occur [5]. And hydrogen has an explosion limit of 4–75%, meaning that any hydrogen leak has a high risk of causing an explosion. Therefore, developing a sensor that can selectively detect hydrogen content in the environment is of great significance for the safer application of hydrogen energy [6]. Metal oxide semiconductors (MOSs), such as TiO_2_, ZnO, WO_3_, In_2_O_3_, SnO_2_, etc., have been widely studied as core hydrogen sensitive materials for hydrogen sensors [7,8,9,10,11,12]. SnO_2_ is a typical N-type semiconductor with a bandgap width of 3.6 eV. SnO_2_-based hydrogen sensors have attracted much attention due to their low cost, excellent chemical durability, and simple preparation process [13,14,15]. In addition, the abundant oxygen vacancies in SnO_2_ nanomaterials give them excellent conductivity properties [2]. Based on these characteristics, SnO_2_ nanomaterials have been widely used in gas sensors. There are many methods to prepare SnO_2_ nanomaterial hydrogen sensors, mainly including magnetron sputtering, atomic deposition, sol-gel, and hydrothermal methods. Among them, the hydrothermal method has high purity and a uniform particle size, which is suitable for preparing SnO_2_ nanomaterial hydrogen sensors.

However, pure SnO_2_ nanomaterials have problems such as a long response/recovery time, high operating temperature, and poor selectivity [16,17]. Therefore, in recent years, a large number of scholars have adopted various methods to improve these problems, such as material microstructure design, precious metal composites, heterojunction generation, and doping modification [18,19,20,21,22]. Scholars have modified it by adding graphene. The unique 2D structure of graphene makes it highly sensitive to its surrounding environment, and its surface adsorption properties can enable the application of sensor materials [23]. Ch. Seshendra Reddy et al. [24] found that electrospun derived Al-doped SnO_2_ embedded by graphene nanotubes has a high response and low response/recovery time. Li et al. [25] used a Au_x_Sn intermetallic compound, modifying the reduced graphene oxide (rGO) modified SnO_2_ (Au_x_Sn-rGO-SnO_2_) to detect H_2_. They found that Au_x_Sn-rGO-SnO_2_ nanocomposites showed a rapid and high response to H_2_. In addition, many studies have shown that doping modification is an effective method to improve the response, selectivity, optimal operating temperature, and response/recovery time of SnO_2_-based sensors. Doping is a technique of introducing impurity elements during material synthesis and preparation, which can change the morphology of materials or introduce catalysts to promote reactions, thereby improving the response of sensors and enhancing their performance [26,27,28]. This doping method can change the crystal structure of the material, introduce impurity energy levels, and increase oxygen vacancies, thereby accelerating the response and adsorption (desorption) rate of the sensor, and optimizing the sensing characteristics [2,26,29,30,31]. Singh et al. [32] found that SnO_2_ sensors doped with 3% Er exhibited stronger sensor response and temperature-dependent selectivity toward ethanol and hydrogen. David E. Motaung et al. [33] reported that a CeO_2_-SnO_2_ mixed oxide heterostructure exhibits a good response to H_2_ gas, high sensitivity, good repeatability, and selectivity. They believe that the n–n heterojunction formed between CeO_2_ and SnO_2_ is the main reason for its improved performance.

According to previous research, the composite material formed by adding 4 mg SLG and SnO_2_ has the best performance [34]. Therefore, this article studied the effect of doping different amounts of Ce in SnO_2_/SLG-4 mg substrate material on hydrogen sensing performance. It was found that the hydrogen sensitivity of the material was optimal when the doping amount of Ce was 2 mol%. The main reason for the enhanced hydrogen sensitivity performance is the coexistence of a n–n heterojunction and n–p heterojunction in doped materials, which increases the carrier migration rate, grain refinement, oxygen vacancy concentration, and the increase in active sites caused by graphene.

## 2. Materials and Methods

### 2.1. Synthesis of S-4G

**Figure 1:**
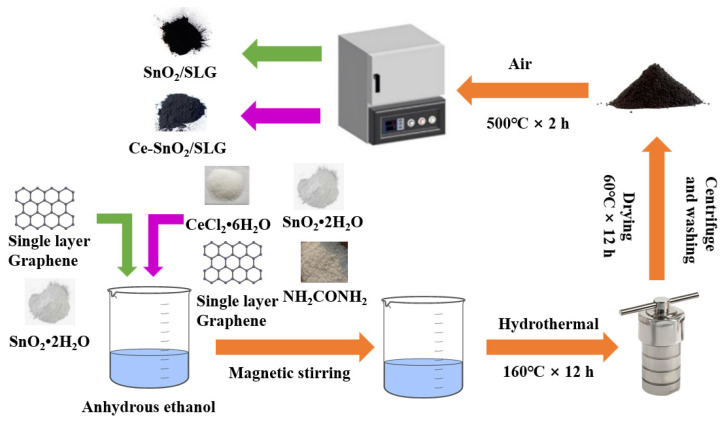
Synthetic scheme of the samples.

SnO_2_/SLG were prepared using a hydrothermal method. The detailed preparation process is shown in Figure 1. Firstly, 1.02 mmol of SnCl_2_·2H_2_O, 4 mg of SLG, and 12.45 mmol of H_2_NCONH_2_ powder were weighed. The weighed powder was mixed and poured into 60 mL of anhydrous ethanol, sonicated for 10 min, and then stirred with a magnetic stirrer for 30 min. The stirred solution was transferred to a reaction vessel and reacted at 16 °C for 12 h. The final product was collected by centrifugation and washed with anhydrous ethanol six times. Afterward, the completely dry white powder was placed at 500 °C for 2 h to obtain SnO_2_/SLG samples, labeled S-4G.

### 2.2. Synthesis of S-4G-xC

xCe-SnO_2_/SLG (x = 1, 2, 3 mol%) were prepared using the hydrothermal method. The detailed preparation process is shown in Figure 1. Firstly, 1.02 mmol of SnCl_2_·2H_2_O, x mol% of CeCl_2_·6H_2_O (x = 1, 2, 3), 4 mg of SLG, and 12.45 mmol of H_2_NCONH_2_ powder were weighed. The weighed powder was mixed and poured into 60 mL of anhydrous ethanol, sonicated for 10 min, and then stirred with a magnetic stirrer for 30 min. The stirred solution was transferred to a reaction vessel and reacted at 160 °C for 12 h. The final product was collected by centrifugation and washed with anhydrous ethanol six times. Afterward, the completely dry white powder was placed at 500 °C for 2 h to obtain xCe-SnO_2_/SLG (x = 1, 2, 3 mol%) samples, labeled S-4G-xC (x = 1, 2, 3).

### 2.3. Characterization

The X-ray diffraction (XRD) pattern of the synthesized samples was obtained from the D/MAX-2200V (Riken Electric Co., Ltd., Tokyo, Japan) diffractometer system with Cu-Kα radiation in the scanning range of 20–90 degrees. Room-temperature Raman spectra of fabricated samples were acquired using a Microconfocal Raman spectrometer from ‘InVia Qontor, Renishaw Ltd., Wotton-under-Edge, UK’ with an excitation wavelength of 532 nm. The surface morphology and X-ray energy-dispersive spectroscopy (EDS) of the samples were recorded using a field emission scanning electron microscope (FESEM) from Japan Electronics JSM-IT800 (JEOL Ltd., Akishima, Japan). The transmission electron microscope (TEM) micrographs of samples have been obtained by means of ‘JEM-F200′ (JEOL Ltd., Akishima, Japan) operating at 200 keV. The X-ray photoelectron spectroscopy (XPS) spectrum of the sample was obtained from an X-ray photoelectron spectrometer manufactured by Thermo Fisher Scientific in the Loughborough, UK, using an Al-K_α_ monochromatic X-ray spectrum.

### 2.4. Fabrication and Performance Test of the Gas Sensor

**Figure 2:**
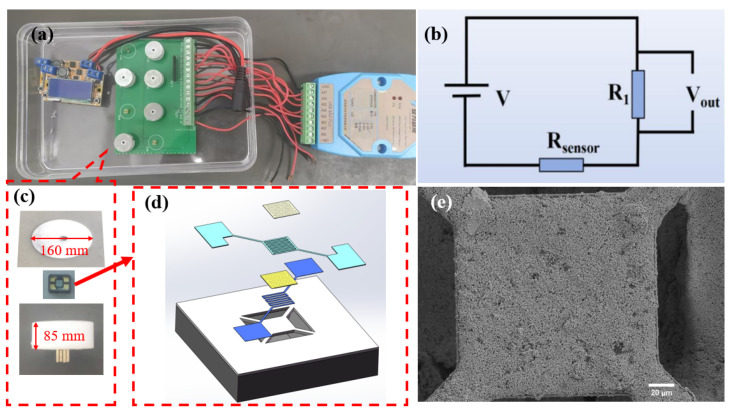
(**a**) Full device diagram of the gas sensitivity performance testing device; (**b**) circuit schematic diagram; (**c**) assembly diagram of the gas sensing element; (**d**) assembly diagram of gas-sensitive elements; (**e**) SEM image of the sensitive layer.

In this work, we utilized HHC1000 micro-hotplate gas-sensitive components provided by Micro & Nano Sensing Technologies Co., Ltd. (Hefei, China). As shown in Figure 2, these components are manufactured based on silicon microelectromechanical systems (MEMS) technology, specifically designed for the fabrication of metal oxide semiconductor (MOS) gas sensors. Featuring a suspended membrane structure, they are characterized by low power consumption and high reliability. The structure mainly includes interdigitated electrodes (IDE), an isolation membrane, a heater, and a support membrane. The IDE and sensing material form the sensing layer, which reacts with the target gas and converts changes in gas concentration into measurable electrical signals. The isolation membrane prevents electrical connections between the heater and the IDE. The micro heater provides the necessary heating conditions for gas sensing, while the support membrane offers structural support to the sensitive area, ensuring the device’s strength. The IDE leads are connected to a four-probe metal setup, which is encapsulated for sensing tests and data acquisition. The IDE is a flexible electrode with Pt.

Before conducting gas sensing tests, the sensing material needs to be integrated into the micro-hotplate gas-sensitive component to form the sensor. The preparation process is as follows: a suitable amount of hydrogen-sensitive material and anhydrous ethanol are placed in an agate mortar and ground into a suspension. Using an ultra-fine paintbrush, a drop of the suspension is applied to the IDE area of the micro-hotplate gas-sensitive component to form a sensing film. After the suspension dries, the gas-sensitive component is heated and aged on a gas sensitivity test bench at 250 °C for one day to promote material deposition and performance stabilization, thereby ensuring reliable gas sensitivity data.

Gas sensitivity testing is performed on the intelligent analysis system of the Yawei Electronic Gas Sensitivity Tester, as depicted in Figure 2a. The measurement circuit for the gas sensor is illustrated in Figure 2b. The temperature (working temperature) of the heater at the chip’s center is controlled by adjusting the heating voltage. The gas response is tested using a steady-state gas distribution method. During the testing process, a microinjector is used to introduce the required amount of target gas. In the circuit for measuring gas response, a load resistor (R_1_) is connected in series with the MEMS gas sensor. The output voltage (V_out_) is recorded 10 times per second. The relationship between the MEMS sensor’s resistance (R) and V_out_ is shown in Equation (1):

R_sensor_ = (V − V_out_) R_1_/V_out_ (1)

This testing equipment features 8 channels for simultaneous testing of 8 sensor units. Each sensor unit comprises a base, a gas sensing element, and a bottom cover, as depicted in Figure 2c. The base transmits the electrical signals from the gas sensing element to the testing circuit board, enabling rapid collection of the changing signals. The gas-sensing element, pivotal to sensors, detects changes in hydrogen concentration in the external environment, resulting in resistance variations. The assembly diagram of gas sensing element is shown in Figure 2d. A magnetic attraction between the bottom cover and base stabilizes the gas sensing element, enhancing signal stability. When assessing the gas sensing capabilities of these sensors, they are preheated to their operating temperature until their resistance stabilizes. Figure 2e presents a SEM image of the sensitive layer, measuring 150 µm in length and 150 µm in width.

The time taken by the sensor to achieve 90% of the total resistance change was defined as the response time in the case of adsorption or the recovery time in the case of desorption. The response of the sensor was measured between 190 and 350 °C. The sensor response to the gas is defined as R_a_/R_g_. R_a_ is the resistance in the air, and R_g_ is the resistance in the target gas. Gas sensitivity tests were conducted under different concentrations of H_2_ conditions. The entire experiment was conducted under a humidity of 20% and monitored using an analysis system. The carrier gas for the sensing measurements is air.

## 3. Results and Discussion

### 3.1. X-ray Diffraction

**Figure 3:**
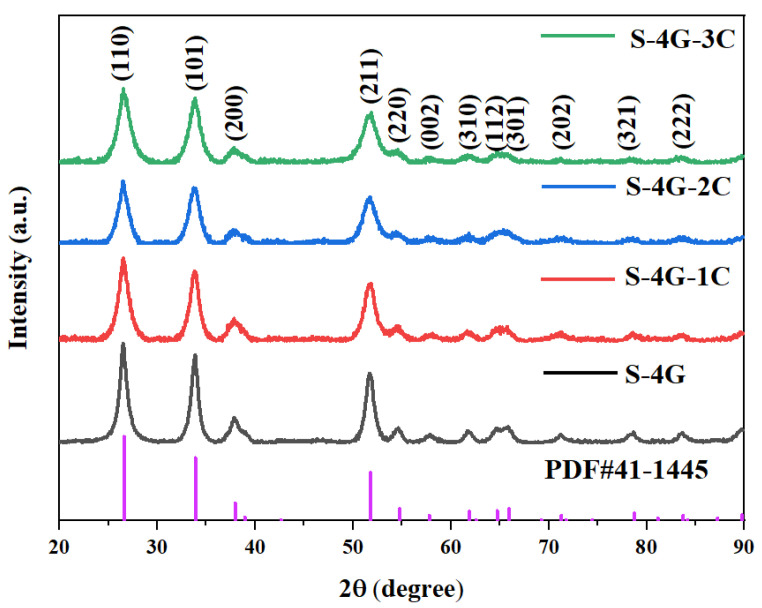
XRD patterns of undoped and Ce-doped SLG/SnO_2_ nanoparticles.

Figure 3 displays the XRD patterns of undoped and Ce-doped SLG/SnO_2_ nanoparticles. The sample’s diffraction peaks align with the standard diffraction peaks of SnO_2_ (PDF # 41-1445), suggesting that the concurrent doping with cerium does not harm or modify the crystal structure of SnO_2_. Moreover, the derived material possesses a pure cubic rutile structure. The crystallite size and lattice constant of all the samples were estimated by using Scherrer’s formula:

(2) D=0.9λ/βcosθ

where *λ* is the wavelength of incident X-rays, *θ* is the Bragg’s angle, *β* is the full width at a half maximum.

The calculated values of crystallite size of undoped and Ce-doped SLG/SnO_2_ nanoparticles are presented in Table 1. It is clear from tabulated values that crystallite size has reduced with an increase in Ce content which indicates that dopant suppressed the growth of SLG/SnO_2_ nanocrystallites. The smaller size can stimulate the quantum tunneling effect, which greatly promotes the gas-sensitive performance growth [35].

### 3.2. Scanning Electron Microscopy

**Figure 4:**
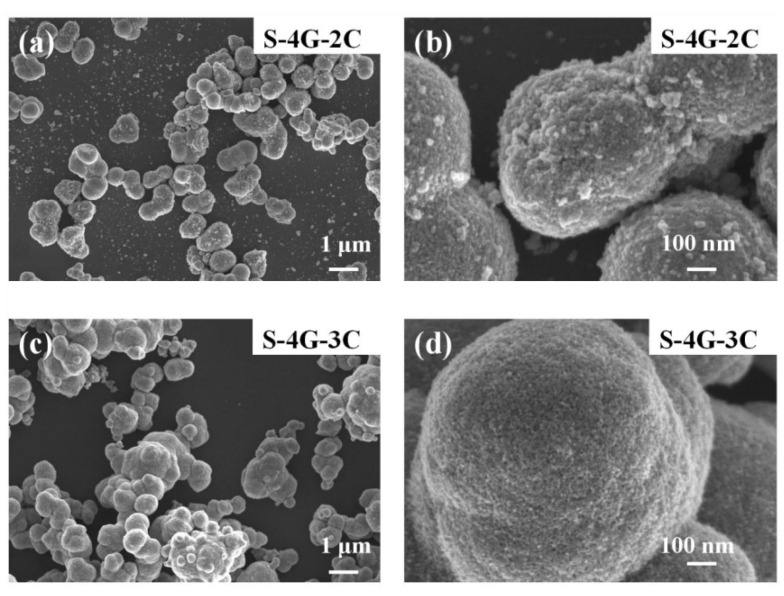
SEM image of (**a**) S-4G-2C; (**b**) S-4G-2C at a higher magnification; (**c**) S-4G-3C sample; (**d**) S-4G-3C at a higher magnification.

Figure 4a–d are SEM images of S-4G-2C and S-4G-3C materials, respectively. It can be seen that both S-4G-2C and S-4G-3C materials have nanosphere structures, and the diameter of the nanosphere structure in S-4G-2C is about 610 nm, while the diameter of the nanosphere structure in S-4G-3C is about 800 nm. At the same time, a certain degree of agglomeration phenomenon occurs, and the agglomeration phenomenon in S-4G-3C is relatively more severe. Therefore, S-4G-2C has a larger specific surface area compared to S-4G-3C. And from Figure 4b,d, it can be seen that the spherical surface of S-4G-2C is rougher, which can provide more active sites, facilitate the adsorption of more gases, and improve the gas sensing performance of the material. Therefore, an excessive addition of Ce leads to severe aggregation of Ce-SnO_2_/SLG nanosphere materials, resulting in a decrease in specific surface area and active sites on the surface, thereby reducing the hydrogen sensitivity of the material [36,37].

### 3.3. Transmission Electron Microscopy

**Figure 5:**
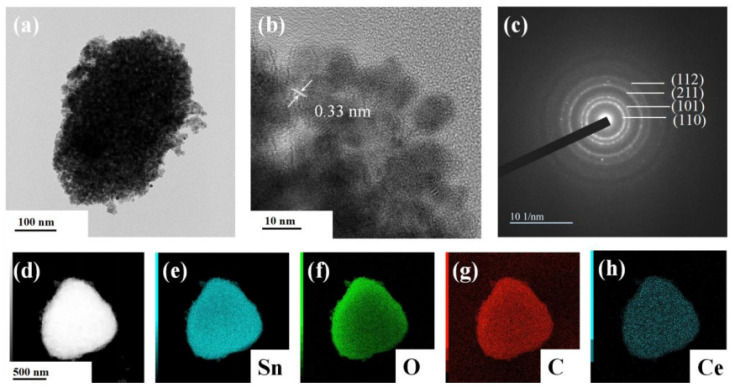
(**a**) TEM image; (**b**) HRTEM image; and (**c**) selected area electron diffraction image of S-4G-2C sample; (**d**–**h**) EDS spectra of the S-4G-2C sample: (**d**) selected region of the energy spectrum, (**e**) Sn element energy spectrum, (**f**) O element energy spectrum, (**g**) C element energy spectrum, and (**h**) Ce element energy spectrum.

The structural properties of synthesized nanoparticles were further studied using transmission electron microscopy. It can be seen that SnO_2_ nanocrystallines were coated on the surface of the SLG material, with an average particle size of about 6.79 nm. The lattice stripe of SnO_2_ NPs can be clearly seen from the Figure 5b HRTEM image, with a lattice spacing of 0.33 nm, corresponding to the rectangular rutile type (110) surface. As shown in Figure 5c, a series of diffraction rings belonging to SnO_2_ can be seen in the selective electron diffraction pattern, which further shows that the prepared SnO_2_ is of the rectangular rutile type and has a polycrystalline structure. The EDS image of S-4G-2C is shown in Figure 5d–h. It can be seen from the figure that Sn, O, C, and Ce elements are uniformly distributed on S-4G-2C, which also proves the successful preparation of the Ce-SnO_2_/SLG material.

### 3.4. X-ray Photoelectron Spectroscopy

**Figure 6:**
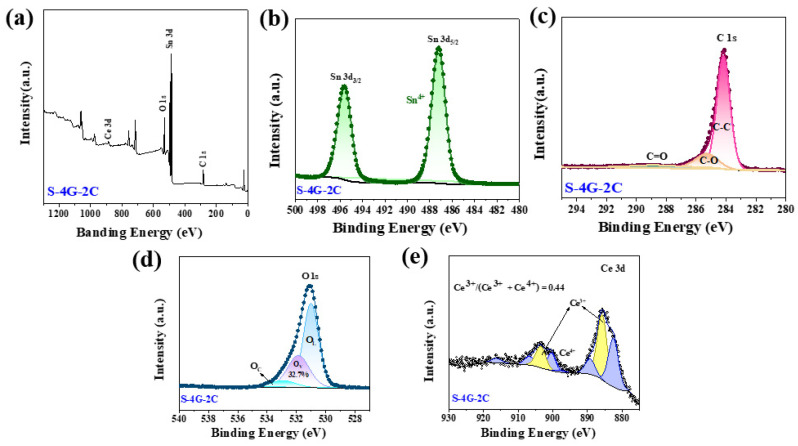
(**a**) XPS full spectrum of the S-4G-2C samples; high-resolution XPS spectra of (**b**) Sn 3 d, (**c**) C 1s, (**d**) O 1s, and (**e**) Ce 3d.

Figure 6a shows the XPS full spectrum of S-4G-2C, where Sn 3d, C 1s, O 1s, and Ce 3d can be observed. Figure 6b shows the high-resolution XPS spectra of Sn 3d, where the Sn 3d_5/2_ peak appears at 486.7 eV and the Sn 3d_3/2_ peak is located at 495.1 eV, indicating that the chemical state of Sn is +4 valence. Figure 6c shows the high-resolution spectrum of C 1s in the S-4G-2C sample. It can be seen that it mainly has three characteristic peaks, located at 284.2 eV, 285.3 eV, and 288.6 eV, respectively, belonging to aromatic carbon (C-C), epoxy and alkoxy carbon (C-O), and hydroxyl carbon (C=O). The introduction of C=O and C-O indicates the formation of sp^3^ hybridization in the SLG structure, and the introduction of sp^3^ hybridization bonds will inevitably weaken sp^2^ hybridization, and increase disorders and defects, which is conducive to increasing the surface-active sites of the material. As shown in Figure 6d, the high-resolution spectrum of O 1s can be divided into three peaks, corresponding to lattice oxygen (O_L_), oxygen vacancies (O_V_), and chemisorbed oxygen (O_C_), respectively.

**Figure 7:**
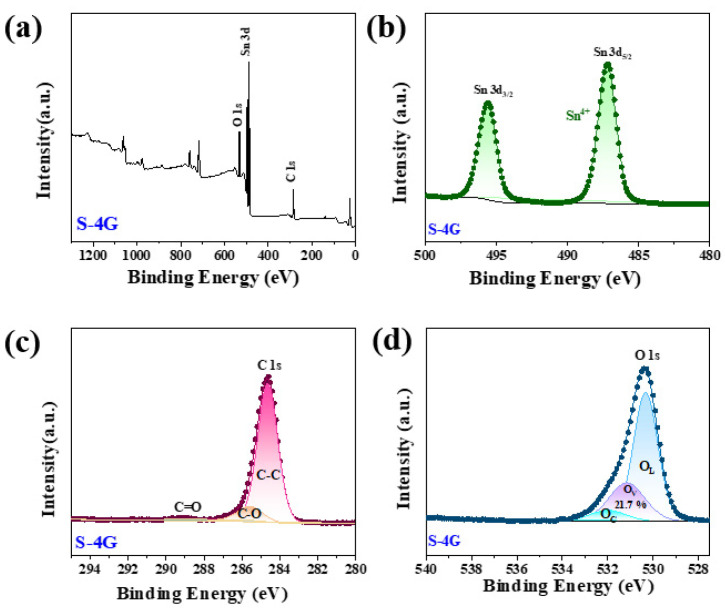
(**a**) XPS full spectrum of the S-4G samples; high-resolution XPS spectra of (**b**) Sn 3 d; (**c**) C 1s; (**d**) O 1s.

Figure 7a shows the full spectrum of sample S-4G, indicating that the sample consists of Sn, O, and C elements. Figure 7b shows the high-resolution map of Sn 3d, in which the Sn 3d_5/2_ peak appears at 487.2 eV and the Sn 3d_3/2_ peak at 495.6 eV, indicating that the chemical state of Sn is +4 valence. Figure 7c shows the high-resolution map of C 1s in the S-4G sample. As can be seen from the figure, it mainly has three characteristic peaks at 284.6 eV, 285.6 eV, and 288.9 eV, belonging to the aromatic carbon (C-C), epoxy and alkoxy carbon (C-O), and hydroxyl carbon (C=O). The peak of C=O and C-O indicates that the sp^3^ hybrid is generated in the SLG structure [38]. The high-resolution map of Figure 7d can be divided into three peaks, corresponding to lattice oxygen (O_L_, 529.0–529.5 eV), oxygen vacancy (O_V_, 530.9–531.2 eV), and chemisorbed oxygen (O_C_, 532.3–533.1 eV). Previous reports in the literature indicate that the surface oxygen state of most chemically resistive sensors has a great influence on their gas sensing performance.

The O_V_ ratio can effectively reflect the concentration of surface oxygen vacancies. It was found that the concentration of surface oxygen vacancies follows the order of S-4G-2C (32.7%) > S-4G (21.7%), and the response also follows the order of S-4G-2C (2.49) > S-4G (1.98), indicating that the concentration of oxygen vacancies greatly affects the response of the material. This is because abundant O_V_ generates a large number of free electrons to maintain high carrier mobility, which is conducive to improving the gas sensing performance of the material. The interaction between Ce and SLG is beneficial for increasing the oxygen vacancy content of the sample, thereby greatly improving the hydrogen sensing performance of the material.

### 3.5. Raman Spectroscopy

**Figure 8:**
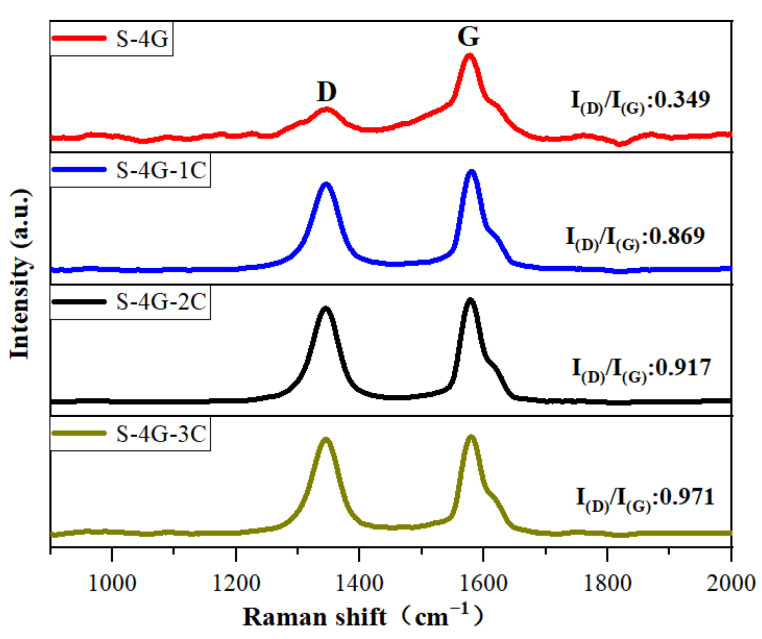
Raman spectra of the S-4G, S-4G-1C, S-4G-2C, and S-4G-3C samples.

Raman spectroscopy is usually used to investigate the crystal defects, crystal structure, crystallinity, and size effects of oxide semiconductors. Figure 8 exhibits the Raman spectra of S-4G, S-4G-1C, S-4G-2C, and S-4G-3C samples. The D peak near 1350 cm^−1^ represents the crystal defects of carbon atoms, and the G peak near 1580 cm^−1^ represents the stretching vibration within the sp^2^ hybridization plane of carbon atoms. I_(D)_/I_(G)_ can reflect the degree of disorder in the sp^2^ carbon domain within the graphene plane [39,40]. I_(D)_/I_(G)_ can be used to evaluate defects in materials, and a larger I_(D)_/I_(G)_ indicates more crystal defects in carbon materials. According to Raman calculations, S-4G-3C (I_(D)_/I_(G)_ = 0.971) > S-4G-2C (I_(D)_/I_(G)_ = 0.917) > S-4G-1C (I_(D)_/I_(G)_ = 0.869) > S-4G (I_(D)_/I_(G)_ = 0.349). As the Ce content increases, the sample becomes more disordered and has more crystal structure defects, making the gas in the material easy to adsorb but difficult to desorb, resulting in a longer recovery time than the response time.

### 3.6. Gas Sensing Properties

**Figure 9:**
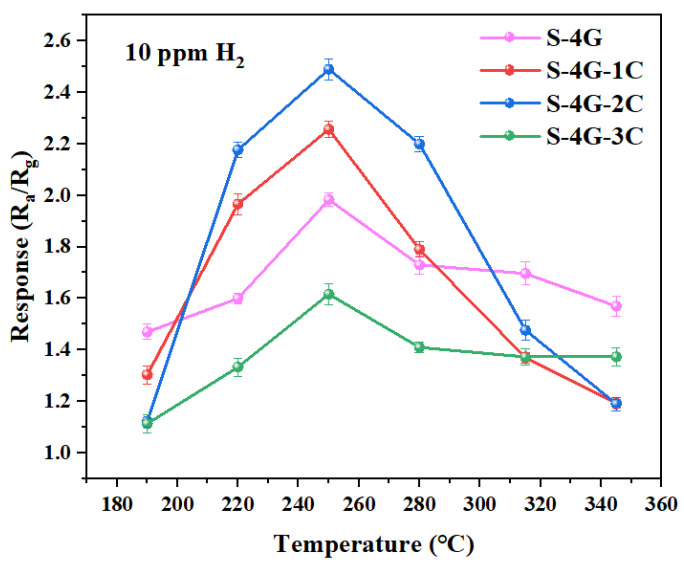
Response of the S-4G and S-4G-xC (x = 1, 2, 3) sensors to 10 ppm of H_2_ at different temperatures.

The optimal operating temperature and response is the major indicators for evaluating gas sensors, and their testing occupies the first priority in all gas sensitivity tests. Therefore, it is particularly important to determine the optimal operating temperature test, which plays a vital role in evaluating the performance of the gas sensor. In order to determine the optimal operating temperature of SnO_2_, S-4G, S-4G-1C, S-4G-2C, and S-4G-3C from 190 °C to 345 °C, these samples were tested for different temperature responses of 10 ppm hydrogen (each set of experiments was repeated three times), as shown in Figure 9. The specific values are listed in Table 2. It can be seen that S-4G, S-4G-1C, S-4G-2C, and S-4G-3C all reach the maximum response at 250 °C, which are 1.98, 2.25, 2.49, and 1.61, respectively. The response of the dual-doped samples S-4G-1C and S-4G-2C at the optimal operating temperature increased by 13.6% and 25.75% compared to S-4G. And the S-4G-3C decreased by 18.69%.

S-4G-2C reached the maximum response (2.49) of the whole system at 250 °C, namely, 2% Ce-SnO_2_/SLG-4 mg was the optimal sample. It can be seen that the hydrogen-sensitive response of Ce-SnO_2_/SLG was significantly higher than that of SnO_2_/SLG sample. The higher response of doping is due to the good synergy among P-type semiconductor SLG material, N-type metal oxide semiconductor SnO_2_, and N-type metal oxide semiconductor CeO_2_. Compared with SnO_2_/SLG, there are three heterojunction types: SLG, SnO_2_, CeO_2_, and the structural complexity of the material is significantly improved.

**Figure 10:**
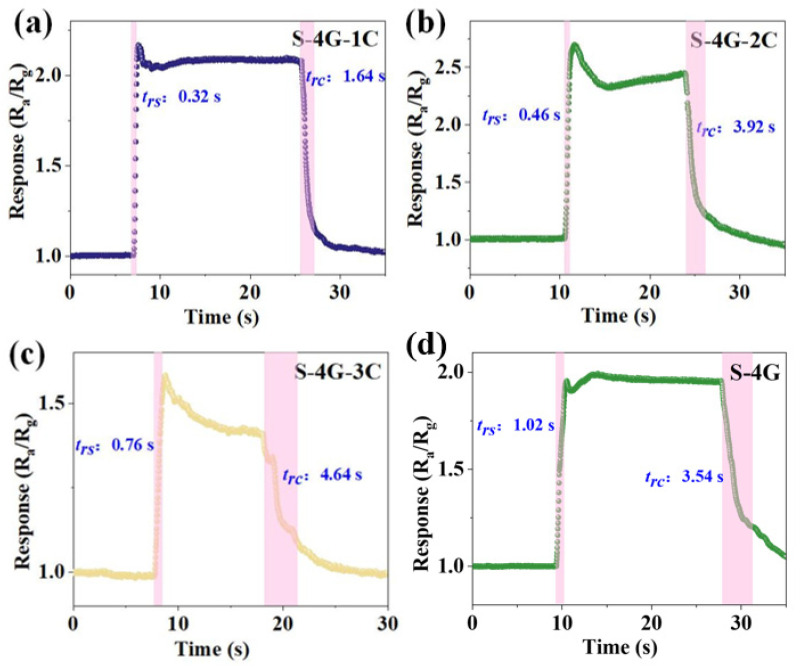
Response/recovery times for 10 ppm hydrogen at an operating temperature of 250 °C for (**a**) S-4G-1C; (**b**) S-4G-2C; (**c**) S-4G-3C; and (**d**) S-4G.

The response (t_rs_)/recovery (t_rc_) time of S-4G and S-4G-xC (x = 1, 2, 3) at 250 °C was tested. As shown in Figure 10a–d, the response time of the doped sample (S-4G-1C 0.32 s; S-4G-2C: 0.46 s; S-4G-3C: 0.76 s) was significantly reduced from S-4G (1.02 s). The recovery time was significantly much greater than the response time. This may be related to the fact that graphene is prone to gas adsorption but difficult to desorb, and when gas molecules adsorb on the material surface, they usually release heat. When gas molecules desorb from the material surface, they need to absorb heat. The difference in energy changes during this adsorption and desorption process leads to a faster response speed of the material in gas detection applications, while the recovery speed may be relatively slow.

**Figure 11:**
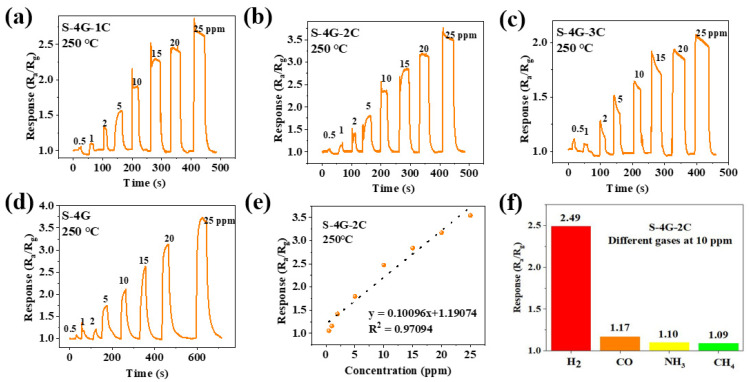
Response curves of (**a**) S-4G-1C; (**b**) S-4G-2C; (**c**) S-4G-3C; (**d**) S-4G at 250 °C under different H_2_ concentrations. (**e**) Fitting curve of the response of S-4G-2C as a function of H_2_ concentration. (**f**) Response of S-4G-2C to different gases (10 ppm) at 250 °C.

Figure 11a–d shows the response curves of the S-4G and S-4G-xC samples (x = 1, 2, 3) at 250°C under different gas concentrations. The response of the test sample varied with the gas concentration, and the response always increased with the increase in gas concentration. The detection limit of the doped SLG/SnO_2_ was 0.5 ppm. As shown in Figure 11e, the fitting curve of the sensor response is plotted with gas concentration as the horizontal axis and response as the vertical axis, reflecting the linear relationship between response and hydrogen concentration. It can be seen that the S-4G-2C sample has a positive linear relationship, which allows for a better estimation of gas concentration in the environment based on the sensor response.

The selectivity of the sensor toward different interfering gases was tested. The response of the S-4G-2C sample to different gases such as hydrogen, carbon monoxide, ammonia, and methane at 10 ppm were tested at 250 °C. The results are shown in Figure 11f. The response of S-4G-2C samples to hydrogen was higher than those of the other gases. Ce-doped samples exhibited better selectivity toward hydrogen gas.

**Figure 12:**
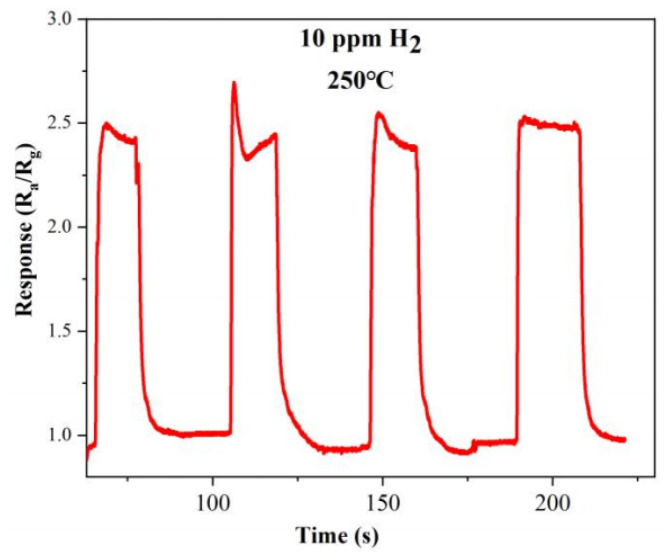
Repeatability of S-4G-2C in a 10 ppm hydrogen atmosphere at 250 °C.

The two other important indicators for evaluating the performance of gas sensors are repeatability and stability. The dynamic response curve of the optimal doped sample S-4G-2C over four test cycles at a hydrogen concentration of 10 ppm at 250 °C is shown in Figure 12. From the figure, it can be seen that the gas sensing performance of the sample is relatively stable and the response did not significantly decreased, indicating that the sample has good stability and repeatability.

Additionally, the performance of the optimally doped sample S-4G-2C was compared to the other reported SnO_2_-based sensors for hydrogen gas sensing (Table 3). It is evident that the sensor co-doped with rare earth Ce and single-layer graphene materials exhibits an extremely rapid response and recovery time, as well as a high response for hydrogen gas detection at relatively lower operating temperatures, confirming that Ce-SnO_2_/SLG sensors have good application prospects and are suitable for hydrogen gas detection.

### 3.7. Sensing Mechanism

It is generally accepted that the sensing response arises from changes in the resistance of gas sensors, induced by the adsorption and desorption of gas molecules on the material surface. The detailed sensing mechanism has been described in references [43,44]. The oxygen vacancies play a critical role in the sensing performance. At different temperatures, oxygen molecules will chemically adsorb on the surface of SnO_2_ in the form of O_2_^−^, O^−^, and O^2−^:

O_2_ (gas) → O_2_ (ads) (3)

O_2_ (ads) + e^−^ → O_2_^−^ (ads) (4)

O_2_^−^ (ads) + e^−^ → 2O^−^ (ads) (5)

O^−^ (ads) + e^−^ → O^2−^ (ads) (6)

Moreover, the oxygen species that have been adsorbed can react with H_2_ molecules on the surface, following the equation provided below:

H_2_ + 1/2 O_2_^−^ (ads)→ H_2_O (ads) + e^−^ (7)

H_2_ + O^−^ (ads) → H_2_O (ads) + e^−^ (8)

The interaction between the reducing gas and oxygen results in a reduction in adsorbed oxygen, which in turn reverses the band bending and enhances conductivity. SnO_2_ is an N-type semiconductor, thereby its conductivity is primarily influenced by the quantity of oxygen adsorbed on its surface. Oxygen ions are formed as oxygen absorbs electrons from the conduction band of tin oxide, leading to the creation of vacancies and subsequently high resistance. When SnO_2_ nanoparticles are exposed to H_2_, the chemisorbed oxygen anions on the surface of tin oxide react with hydrogen, leading to the removal of chemisorbed oxygen anions and the oxidation of H_2_ molecules. Consequently, the free electrons captured by these oxygen molecules are released back to the conduction band of tin oxide, resulting in a decrease in resistance.

The presence of both Ce^3+^ and Ce^4+^ on the surface of Ce-doped SnO_2_ hydrogen-sensitive materials generates a significant number of additional oxygen vacancies. Ce^4+^, being an oxidizing ion, readily captures electrons, resulting in the formation of Ce^3+^ ions and the concomitant creation of numerous oxygen vacancies [45]. Moreover, at the interface of Ce-SnO_2_/SLG composites, multiple heterogeneous interfaces exist. Specifically, an n–n junction forms between CeO_2_ and SnO_2_, and a p–n junction between SnO_2_ and SLG [46]. Because CeO_2_ has a lower work function than SnO_2_, the n–n heterojunction facilitates a greater transfer of electrons from CeO_2_ to SnO_2_. This leads to more holes on SLG moving to SnO_2_, and vice versa, until a dynamic equilibrium is reached. This facilitation thickens the depletion layers between CeO_2_ and SnO_2_, as well as between SnO_2_ and SLG, thereby increasing the material’s resistance in air. In a hydrogen environment, hydrogen molecules react with adsorbed oxygen on the material surface, releasing electrons back into SnO_2_. This reaction diminishes the depletion layer’s depth, reduces the energy band’s curvature, and lowers the potential barrier. Consequently, charge carriers transfer more readily, enhancing the material’s conductivity and significantly reducing its resistance.

## 4. Conclusions

In this work, Ce-SnO_2_/SLG composite materials were prepared using a hydrothermal method, which has the advantages of simple operation, low cost, and the ability to achieve large-scale production. Among them, the 2% Ce-SnO_2_/SLG-4 mg composite material has the best hydrogen sensitivity performance at a humidity of 20%, with a high response rate of 2.49. The reaction time decreased from 1.02 s to 0.46 s, indicating an extremely low response time. When detecting 10 ppm H_2_ at 250 °C, the response and recovery times are 0.46 s and 3.92 s, respectively. Additionally, the sensor exhibits excellent selectivity and reproducibility for hydrogen gas. The reason why doping Ce enhances the hydrogen sensitivity of SnO_2_ can be attributed to the following: an increase in Ce doping leads to increased disorder and more defects in the crystal structure of SLG, favoring the creation of more active sites; and Ce doping inhibits the growth of SnO_2_ nanocrystals. Moreover, Ce doping enhances the concentration of oxygen vacancies (32.7%), thereby increasing active sites and boosting the material’s hydrogen sensitivity. Moreover, Ce doping facilitates the formation of n–n and p–n heterojunctions, which, through their combined effect, enhance charge carrier transfer, increase the material’s depletion layer thickness, and elevate its hydrogen sensitivity.

## Figures and Tables

**Table 1:** Crystallite size of undoped and Ce-doped SLG/SnO_2_ nanoparticles.

Sample	S-4G	S-4G-1C	S-4G-2C	S-4G-3C
Ce content (mol%)	0	1	2	3
Crystallite size from XRD (nm)	9	8	5	6.1

**Table 2:** Response of the SnO_2_, S-4G, and S-4G-xC (x = 1, 2, 3) sensors to 10 ppm of H_2_ at different temperatures.

Sample	Response at Different Temperatures					
	190 °C	220 °C	250 °C	280 °C	315 °C	345 °C
S-4G	1.47	1.60	1.98	1.72	1.70	1.57
S-4G-1C	1.30	1.96	1.25	1.79	1.37	1.20
S-4G-2C	1.12	2.17	2.49	2.20	1.47	1.20
S-4G-3C	1.11	1.33	1.61	1.41	1.36	1.37

**Table 3:** Comparison between the present and previous studies on the H_2_ gas response of different gas sensors.

Material	Working Temperature (°C)	Concentration (ppm)	Response	Response Time (s)	Recovery Time (s)	Ref.
Au-functionalized ZnO-branched SnO_2_ NWs	300	10	13.07	190	351	[41]
SnO_2_ thin film with a Pd island	300	250	28	3	50	[42]
Graphene-loaded Al-SnO_2_ nanotubes	300	100	23.8	2.2	1.4	[24]
S-4G-2C	250	10	2.49	0.46	3.92	This work

## Data Availability

The original contributions presented in the study are included in the article, further inquiries can be directed to the corresponding author.
